# Dysregulated systemic lymphocytes affect the balance of osteogenic/adipogenic differentiation of bone mesenchymal stem cells after local irradiation

**DOI:** 10.1186/s13287-017-0527-0

**Published:** 2017-03-20

**Authors:** Xiaoya Xu, Ruixia Li, Yi Zhou, Qiong Zou, Qiaoling Ding, Jinfeng Wang, Weifang Jin, Guoqiang Hua, Jianjun Gao

**Affiliations:** 1 Department of Radiation Biology, Institute of Radiation Medicine, Fudan University, No. 2094 Xie-Tu Road, Shanghai, 200032 China; 2 Obstetrics and Gynecology Hospital of Fudan University, No. 419 Fangxie Road, Shanghai, 200011 China; 3 Department of Bone Metabolism, Institute of Radiation Medicine, Fudan University, No. 2094 Xie-Tu Road, Shanghai, 200032 China

**Keywords:** Irradiation, Lymphocytes, Bone mesenchymal stem cells (BMSCs), RUNX2, PPARγ

## Abstract

**Background:**

While it is known that irradiation can induce local and systemic bone loss over time, how focal irradiation induces systemic bone complications remains unclear. Immune cells are thought to be crucial to bone homeostasis, and abnormal immune cells lead to serious disruption of bone homeostasis, such as in acute lymphoblastic leukaemia. This disruption primarily occurs due to inhibition of the osteogenic differentiation of bone mesenchymal stem cells (BMSCs).

**Methods:**

In this study, we detected local and systemic bone loss in trabecular bone by micro-computed tomography (micro-CT) and measurement of peroxisome proliferator-activated receptor gamma (PPARγ) and runt-related transcription factor 2 (RUNX2) expression in BMSCs using real-time polymerase chain reaction and western blotting. Additionally, changes in lymphocytes (B cells and CD4^+^ and CD8^+^ T cells) in the peripheral blood and bone marrow were analysed by flow cytometry. BMSC-derived osteoblasts and adipocytes, cultured in osteogenic or adipogenic media or co-cultured with lymphocytes, were detected by BCIP/NBT, Alizarin Red S and Oil Red O staining.

**Results:**

Focal irradiation induced local and systemic bone loss in trabecular bone. Increased PPARγ expression and decreased RUNX2 expression were observed, accompanied by upregulated adipogenesis and downregulated osteogenesis of BMSCs. B cells and CD8^+^ T lymphocytes were increased in the blood and bone marrow after irradiation, while CD4^+^ T lymphocytes were decreased in the blood. Inhibition of RUNX2 expression and reduction of alkaline phosphatase activity and mineralization deposits were observed in lymphocyte-co-cultured BMSCs, accompanied by an increase in PPARγ expression and in the number of lipid droplets.

**Conclusions:**

Focal irradiation induced local and systemic bone loss in trabecular bone. Increased B cells and CD8^+^ T lymphocytes led to systemic bone loss by decreasing BMSC osteogenesis.

## Background

Radiotherapy is an effective anti-cancer strategy combined with surgery and/or chemotherapy and reduces mortality rates [1]. However, radiotherapy also reduces patient quality of life, and long-term side effects of radiotherapy, such as bone complications, begin to emerge in cancer survivors. Specifically, the incidence of bone complications at irradiated sites increases after radiotherapy [2–8]. In addition to the direct effects of radiation on bone, bone complications, including bone fracture, occur outside of irradiated sites [9–13]. However, the mechanism by which systemic bone complications occur after a single irradiation treatment has not been elucidated. Some researchers have suggested that oxidative stress affects bone after irradiation, while others hypothesized that inflammatory factors induce bone loss after radiation. However, few reports discuss changes in immune cells and their relation to systemic bone loss after a single dose of irradiation.

Recent observations that abnormal activation of the immune system can lead to bone destruction and that mice deficient in immunomodulatory molecules often develop an unexpected skeletal phenotype [14–16] have led to the view that there is a close relationship between the bone and immune systems, particularly under pathological conditions [17]. Irradiated areas have damage to almost all tissues (from the skin through to the bone marrow), including local bone and immune cells. Unlike surgery, which generally leads to full recovery, radiation damage lasts for the rest of the patient’s life and may be a source of abnormally activated immune cells that alter bone metabolism.

Here, we used a rat model of focal irradiation to detect changes in microarchitectural structures of bone and B and T lymphocytes in the blood and bone marrow 12 weeks post-irradiation. We also investigated the potential of bone mesenchymal stem cells (BMSCs) to differentiate into osteoblasts and adipocytes after irradiation and observed the effects of lymphocytes from irradiated rats on the differentiation of BMSCs.

## Methods

### Animal treatment

Four-month-old male Sprague-Dawley rats (Shanghai Lab Animal Resource Centre, STCSM, Shanghai, China) were used for the experiments. Rats in the irradiated group (*n* = 40) were anaesthetized with ketamine, placed in a ^137^Cs γ-ray irradiation chamber (MDS Nordion International, Kanata, Canada), and exposed to 20 Gy (0.8 Gy/min for 25 min, ^137^Cs γ-ray irradiation machine) on the right limb in a 2-cm by 2-cm area covering the proximal tibia and distal femur. Unirradiated body parts, including the skeleton, were shielded with a custom-made lead block, and contralateral sides of the femur and tibia (the bone that did not receive radiation but was removed from irradiated rats) represented areas distant from irradiation. Control rats (*n* = 40) were similarly manipulated, anaesthetized, and subjected to sham irradiation (0 Gy). All experiments involving animals were performed according to institutionally approved and current animal care guidelines.

### Micro-CT analysis

Micro-computed tomography (CT) analysis of the tibia was conducted using a SkyScan-1176 micro-CT (μCT, Bruker micro-CT, Kontich, Belgium) system. Scans were performed using a PANalytical Microfocus Tube (17.93-μm voxel size, 65 KV, 385 μA, 0.5-degree rotation step, and 180-degree angular range). Micro-CT evaluation of the trabecular bone was performed on a 2-mm region of metaphyseal spongiosa in the proximal tibia located 0.5 mm above the growth plate. Measurements of the cortical bone were performed on a 1-mm region of the mid-diaphysis of the tibia. NRecon software version 1.6 (Bruker) was used for 3D reconstruction and to view images. After 3D reconstruction, CT software version 1.13 (Bruker) was used for bone analysis.

### Histological examination and histomorphometry

Tibia sections were stained with Oil Red O and Mayer’s haematoxylin and histochemically tested for alkaline phosphatase (ALP) activity using a BCIP/NBT kit (Beyotime Biotechnology, Jiangsu, China) and for tartrate-resistant acid phosphatase (TRAP) activity using a TRACP kit (Sigma-Aldrich, St. Louis, MO, USA). The sections were then counterstained with methyl green and mounted in Kaiser's glycerol jelly. The following parameters were measured: the ALP-positive osteoblast surface per bone surface (OB.S/BS, %) for bone formation, the TRAP-positive osteoclast surface per bone surface (OC.S/BS, %) for bone resorption, and the adipocyte area per bone marrow area without trabecula (%) for marrow adiposity [18]. Images of micrographs from single sections were digitally recorded using a rectangular template, and recordings were processed and analysed using Image-Pro Plus image analysis software (Image-Pro Plus, version 4.112, Media Cybernetics, LP, Silver Spring, MD, USA).

### Measurement of bone turnover markers in serum by ELISA

Blood was collected at 12 weeks post-irradiation. Serum from each rat was analysed individually in duplicate for the bone formation marker osteocalcin (OCN) using a Rat Osteocalcin EIA kit (Immunodiagnostic Systems Inc., Boldon, UK) according to the manufacturer’s instructions. Bone resorption was examined with the marker TRAP 5b (TRAP5b) using a Rat TRAP Assay (Immunodiagnostic Systems Inc.) according to the manufacturer’s instructions. The average value of the duplicate measurements was obtained for each rat.

### Flow cytometry

Blood and bone marrow were collected 12 weeks after radiation. Cells were incubated with anti-rat CD3-FITC/CD45RA-PC7 and anti-rat CD3-FITC/CD4-PC7/CD8-APC (Beckman Coulter, High Wycombe, UK) at room temperature for 20 min, then with OptiLyse C (Beckman Coulter, UK) at room temperature for 10 min. The cells were then washed twice with phosphate-buffered saline (PBS) and resuspended in 500 μl PBS. All samples were assessed by flow cytometry (Beckman Coulter, Brea, CA, USA). The percentage of cells was based on the evaluation of 100,000 events for each culture condition [19].

### Cell culture

BMSCs were flushed from the tibia and femur 12 weeks after irradiation with a-MEM (Gibco BRL, Carlsbad, CA, USA). The cells were seeded on 100-mm culture dishes (Nunc, Rochester, NY, USA) and cultured in L-DMEM supplemented with 100 IU/ml penicillin, 100 mg/ml streptomycin (Gibco BRL), and 10% foetal bovine serum (FBS, Gibco BRL). The media was replaced every 3–4 days to remove non-adherent haematopoietic cells. After 2 weeks, the adherent cells were collected and sorted by flow cytometry with CD29, CD90, and CD34. The sorted BMSCs (CD29+, CD90+, and CD34-) were cultured in fresh medium and further subcultured. The first passage of sorted BMSCs was termed passage 1. BMSCs between passages 3 and 5 were used for experiments. To induce osteogenic differentiation, passage 4 BMSCs were cultured with a-MEM supplemented with 10% FBS, ascorbic acid (50 mg/ml), and b-glycerophosphate (10 mM) for up to 14 days (for ALP staining) or 28 days (for Alizarin Red staining). Adipogenic differentiation was induced by culturing with insulin (10 mg/ml), dexamethasone (1 mM), and 3-isobutyl-1-methylxanthine (0.5 mM) for up to 21 days (for Oil Red O staining).

### Co-culture

Bone marrow mononuclear cells were isolated by density gradient centrifugation (lymphocyte separation medium, Sigma-Aldrich) within 6 h of sampling. Adherent cells were removed using plastic adherent culture dishes, and the remaining cells were immediately co-cultured with normal BMSCs for 3 days. Some samples were used for real-time PCR and western blotting; others were cultured with a-MEM supplemented with 10% FBS, ascorbic acid (50 mg/ml), and b-glycerophosphate (10 mM) for up to 14 days (for ALP staining) and 28 days (for Alizarin Red staining) to induce osteogenic differentiation. Adipogenic differentiation was induced by culturing with insulin (10 mg/ml), dexamethasone (1 mM), and 3-isobutyl-1-methylxanthine (0.5 mM) for up to 21 days (for Oil Red O staining).

### Quantitative real-time PCR

Cultured and co-cultured BMSCs were collected, and total ribonucleic acid (RNA) was extracted using TRIzol reagent (15596; Invitrogen, Carlsbad, CA, USA) according to the manufacturer’s protocol. Total RNA was reverse-transcribed to cDNA using QuantiTect Rev Transcription kits (205311; Qiagen, Chatsworth, CA, USA). The number of cDNA molecules in the reverse-transcribed samples was determined using a modified real-time PCR method with QuantiTect SYBR Green PCR kits (204143, Qiagen) on a Mx3000P Real-Time PCR system (Stratagene, La Jolla, CA, USA). Primers with the following sequences were obtained from SBS Genetech (http://www.sbsbio.com/news/englishnew/index.php) using a previously described protocol: RUNX2, 5'-AGCCTCTTCAGCGCAGTGAC-3' and 5'-CTGGTGCTCGGATCCCAA-3' (132 bp, AF187319); PPARγ, 5'-TCAGGTTTGGGCGAATGC-3' and 5'-TTTGGTCAGCGGGAAGGA3' (152 bp, Nm013124.3); and GAPDH, 5'-AAACCCATCACCATCTTCCA-3' and 5'-GTGGTTCACACCCATCACAA-3' (198 bp, DQ403053) [17]. The PCR reactions included 12.5 μl of Master SYBR Green I, 0.25 μM of each 5' and 3' primer, and 2 μl of samples and/or H_2_O in a final volume of 25 μl. A melting curve was obtained at the end of each run to discriminate specific from nonspecific cDNA products. The cDNA content was normalized by subtracting the cycle numbers of GAPDH from those of the target gene (∆Ct = Ct of target gene – Ct of GAPDH), and gene expression levels were calculated using the 2^–(∆Ct)^ method [20].

### Western blotting analysis

Cultured and co-cultured BMSCs were collected and lysed with radioimmunoprecipitation assay (RIPA) buffer (P0013B; Beyotime Biotechnology, Jiangsu, China). Cells were extracted for 20 min on ice. Insoluble materials were removed by centrifuging at 12,000 g for 30 min. Supernatants were collected, and protein levels were quantified using a bicinchoninic acid assay (BCA) (P0012, Beyotime Biotechnology); bovine serum albumin (BSA) was used as a standard. The sample protein was denatured in boiling water for 5 min in SDS-PAGE sample loading buffer (P0015, Beyotime Biotechnology). Aliquots of samples (40 μg) were then subjected to SDS-PAGE in 12% gels under reducing conditions and electroblotted onto PVDF membranes (Ipvh00010; EMD Millipore, Bedford, MA, USA). The membranes were blocked with 5% fat-free dry milk in Tris-buffered saline and Tween 20 (TBST) (0.1% Tween-20 and 0.1 M NaCl in 0.1 M Tris–HCl, pH 7.5) for 2 h at room temperature. Then, the membranes were incubated at 4 °C overnight with a goat anti-RUNX2 antibody (1:500; ab56326; Abcam, Cambridge, MA, USA), a rabbit anti-PPARγ antibody (1:1500; AT819, Beyotime Biotechnology), or a mouse anti-GAPDH antibody (1:5000; Kangcheng Biotechnology Inc., Shanghai, China). The membranes were then incubated with a horseradish peroxidase-conjugated secondary antibody (1:5000; Santa Cruz Biotechnology, Dallas, TX, USA) at room temperature for 1 h, followed by chemiluminescence detection (P0018, Beyotime Biotechnology). Each incubation step was followed by three washes (10 min each) with TBST. Protein bands were quantitatively analysed using an image analysis system (QuantityOne software; Bio-Rad, Hercules, CA, USA) [20]

### Statistical analysis

Differences were determined by one-way ANOVA with Bonferroni post hoc testing or by paired or unpaired Student’s *t* test, as appropriate (GraphPad Prism 6, version 6.0c, GraphPad Software, San Diego, CA, USA). The results were expressed as the means ± standard derivations, and *P* < 0.05 was considered significant.

## Results

### Bone microarchitecture is changed after a single dose of radiation

**Fig. 1:**
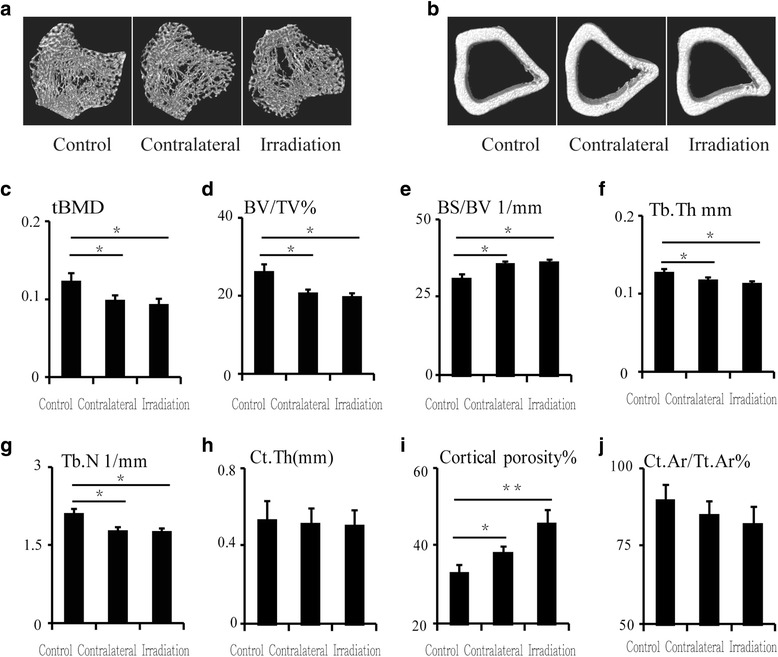
Effects of in vivo radiation exposure to a single bone on trabecular and cortical bone of the tibia 12 weeks post-irradiation. **a**, **b** Representative reconstructed images of μCT scans showing trabecular and cortical bone in the tibia and (**c**) tBMD. Differences in (**d**) BV/TV, (**e**) BS/BV, (**f**) Tb.Th, (**g**) Tb.N, (**h**) Ct.Th, (**i**) cortical porosity, and (**j**) Ct.Ar./Tt.Ar. Data are presented as the means ± standard deviations; ^*^ *P* < 0.05 and ^**^ *P* < 0.01 (*n* = 16/group). *BS/BV* ratio of bone surface to the bone volume, *BV/TV* bone volume fraction, ratio of the segmented bone volume to the total volume of the region of the region of interest, *CT* computed tomography, *Ct.Ar./Tt.Ar* cortical area fraction, *Ct.Th* average cortical thickness, *Tb.N* trabecular number, measure of the average number of trabeculae per unit length, *Tb/Th* trabecular thickness, mean thickness of trabeculae, *tBMD* trabecular bone mineral density

We used μCT to delineate a purely trabecular region of interest that showed changes in bone volume and microarchitectural structure. Twelve weeks post-irradiation (20 Gy), trabecula bone mineral density (tBMD) was reduced by 19.9% (*P* < 0.05) in the contralateral tibia and by 24.4% (*P* < 0.05) in the irradiated tibia relative to the control tibia (Fig. 1a and c). The trabecular bone volume (BV/TV) was reduced by 21.0% (*P* < 0.05) in the contralateral tibia and by 24.8% (*P* < 0.05) in the irradiated tibia relative to the control tibia (Fig. 1d). Bone surface to bone volume (BS/BV) ratio was increased by 15.1% (*P* < 0.05) in the contralateral tibia and by 16.9% (*P* < 0.05) in the irradiated tibia compared with the control tibia (Fig. 1e). Trabecular thickness (Tb.Th) and trabecular number (Tb.N) were significantly reduced at 12 weeks after irradiation. Tb.Th was reduced by 7.6% (*P* < 0.05) in the contralateral tibia and by 11.1% (*P* < 0.05) in the irradiated tibia compared with the control tibia. Meanwhile, Tb.N was reduced by 15.8% (*P* < 0.05) in the contralateral tibia and by 16.5% (*P* < 0.05) in the irradiated tibia relative to the control tibia (Fig. 1f and g). The bone microarchitecture of the cortical tibia changed only slightly, and no significant differences were observed (Fig. 1b). However, cortical porosity increased by 39.3% (*P* < 0.05) and 96.4% (*P* < 0.01) in the contralateral and irradiated tibias, respectively, relative to the control tibias (Fig. 1i).

### Decreased osteoblastogenesis and an increase in adipocytes after a single dose of radiation

**Fig. 2:**
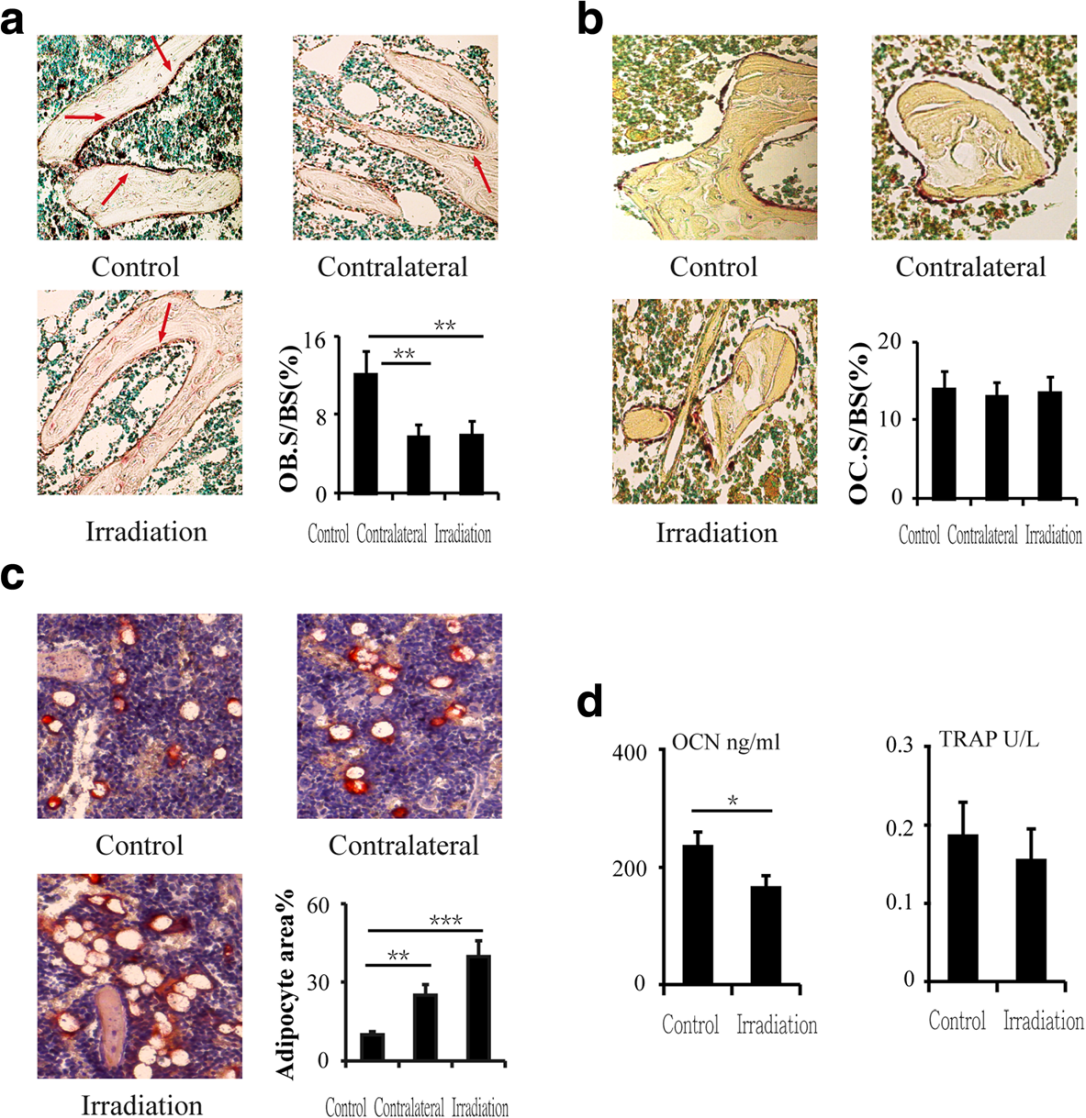
Effects of in vivo radiation exposure to a single bone. **a** ALP-stained sections showing effect on OB.S/BS at 12 weeks. **b** TRAP-stained sections showing effect on OC.S/BS at 12 weeks. **c** Oil Red O-stained sections showing effect on adipocyte area. Data are presented as the means ± standard deviations; ^**^ *P* < 0.01 and ^***^ *P* < 0.001 (*n* = 8/group). **d** Levels of serum OCN and TRAP at 12 weeks. Data are presented as the means ± standard deviations; ^*^ *P* < 0.05 (*n* = 16/group). *ALP* alkaline phosphatase, *OB.S/BS* osteoblast surface to bone surface, *OC.S/BS* TRAP-positive osteoclast surface to bone surface, *OCN* osteocalcin, *TRAP* tartrate-resistant acid phosphatase

To examine changes in osteoblast and osteoclast activity, histomorphometric analysis was performed on ALP- and TRAP-stained sections. The results showed that the ALP-positive osteoblast surface to bone surface (OB.S/BS) decreased by 51.7% (*P* < 0.01) and 50.8% (*P* < 0.01) in the contralateral and irradiated tibias, respectively, relative to the control tibias at 12 weeks after irradiation (Fig. 2a). At this time point, there was no difference in the ratio of TRAP-positive osteoclast surface to bone surface (OC.S/BS) between the two groups (Fig. 2b). ELISA analysis of bone turnover markers in the serum revealed a 29.9% (*P* < 0.05) reduction in OCN at 12 weeks post-irradiation (Fig. 2d); the serum bone resorption marker TRAP5b also tended to be reduced (16.7%, *P* > 0.05) (Fig. 2d). The adipocyte area in the bone marrow of the contralateral and irradiated tibias increased significantly after irradiation, with an increase of 92.6% (*P* < 0.01) in the contralateral tibias and 129.6% (*P* < 0.001) in the irradiated tibias at 12 weeks (Fig. 2c).

### Increasing adipocyte and decreasing osteoblast differentiation of BMSCs in vitro after a single irradiation

**Fig. 3:**
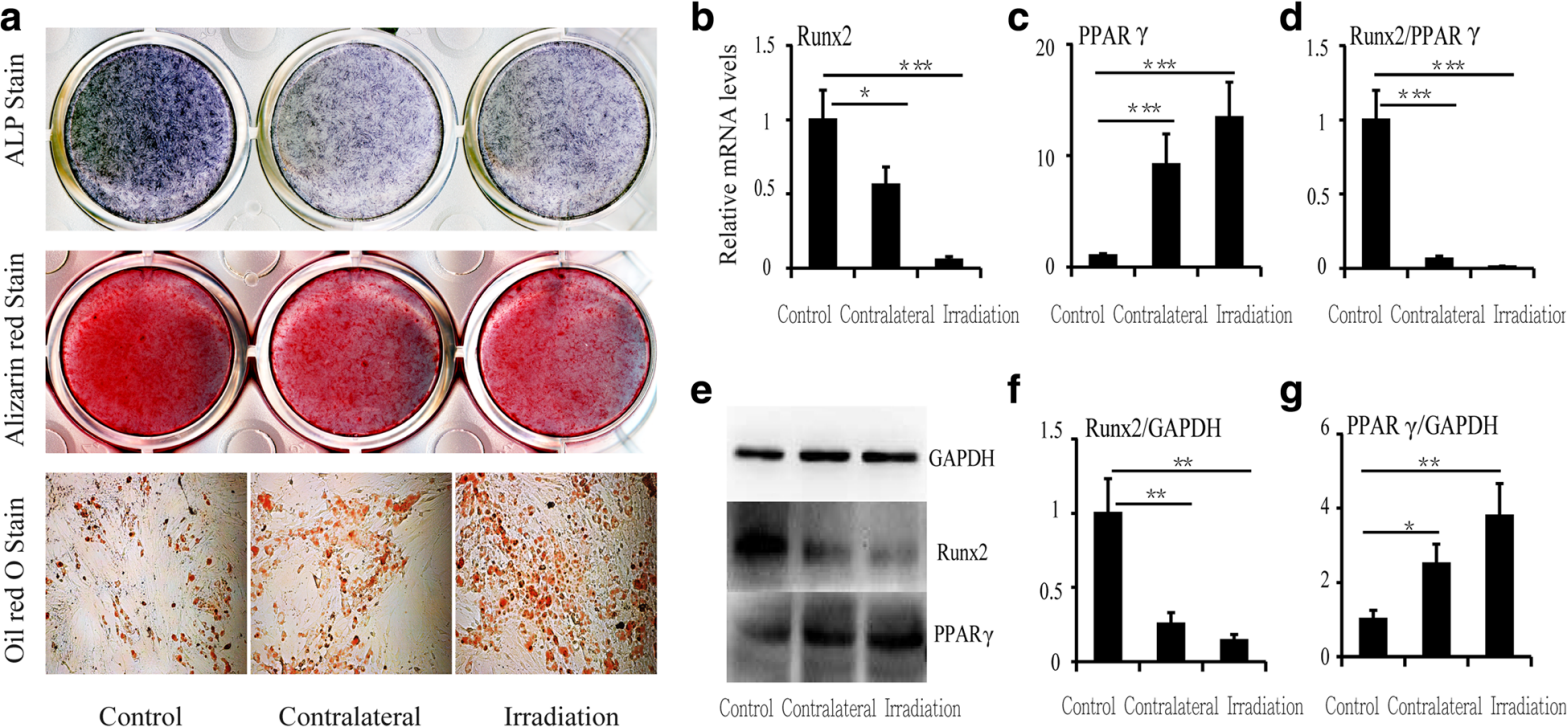
Changes in differentiation of and relative mRNA and protein levels in BMSCs at 12 weeks after irradiation. **a** ALP, Alizarin Red, and Oil Red O staining of BMSCs. **b** RUNX2 mRNA expression, (**c**) PPARγ mRNA expression, and (**d**) RUNX2/PPARγ ratio. Western blot image (**e**) and RUNX2 (**f**) and PPARγ (**g**) protein expression levels at 12 weeks. Data are presented as the means ± standard deviations; ^*^ *P* < 0.05, ^**^ *P* < 0.01 and ^***^ *P* < 0.001 (*n* = 6/group). *ALP* alkaline phosphatase, *PPARγ* peroxisome proliferator-activated receptor γ, *RUNX2* runt-related transcription factor 2

To evaluate the influence of a single irradiation dose on osteoblast/adipocyte differentiation from progenitors, induction cultures of BMSCs were performed. Determination of differentiation was based on a sharp increase in adipocytes and decrease in osteoblasts in bone marrow after irradiation. ALP staining demonstrated that osteoblast differentiation was markedly decreased in cultures from contralateral and irradiated sites relative to controls, and Alizarin Red staining showed the same trend. However, Oil Red O staining increased, demonstrating that adipocyte differentiation was increased after irradiation in cultures from both irradiated and contralateral sites (Fig. 3a).

The expression levels of runt-related transcription factor 2 (RUNX2) and peroxisome proliferator-activated receptor gamma (PPARγ) were determined in BMSCs at 12 weeks after irradiation by real-time PCR and western blotting. There was a significant decrease in RUNX2 mRNA (-44.1% at contralateral sites, *P* < 0.05, and -94.5% at irradiated sites, *P* < 0.001) (Fig. 3b). However, PPARγ expression was markedly upregulated by approximately 9-fold (*P* < 0.001) in contralateral bone and by 13-fold (*P* < 0.001) in irradiated bone relative to controls (Fig. 3c). Therefore, the ratio of RUNX2 to PPARγ decreased sharply, with a 93.9% (*P* < 0.001) reduction in contralateral bone and a 99.0% (*P* < 0.001) reduction in irradiated bone (Fig. 3d). Similar to the corresponding mRNA levels, RUNX2 and PPARγ protein levels were altered at 12 weeks after irradiation (Fig. 3e, f, and g).

### Numbers of lymphocytes in blood and bone marrow were altered after a single irradiation

**Fig. 4:**
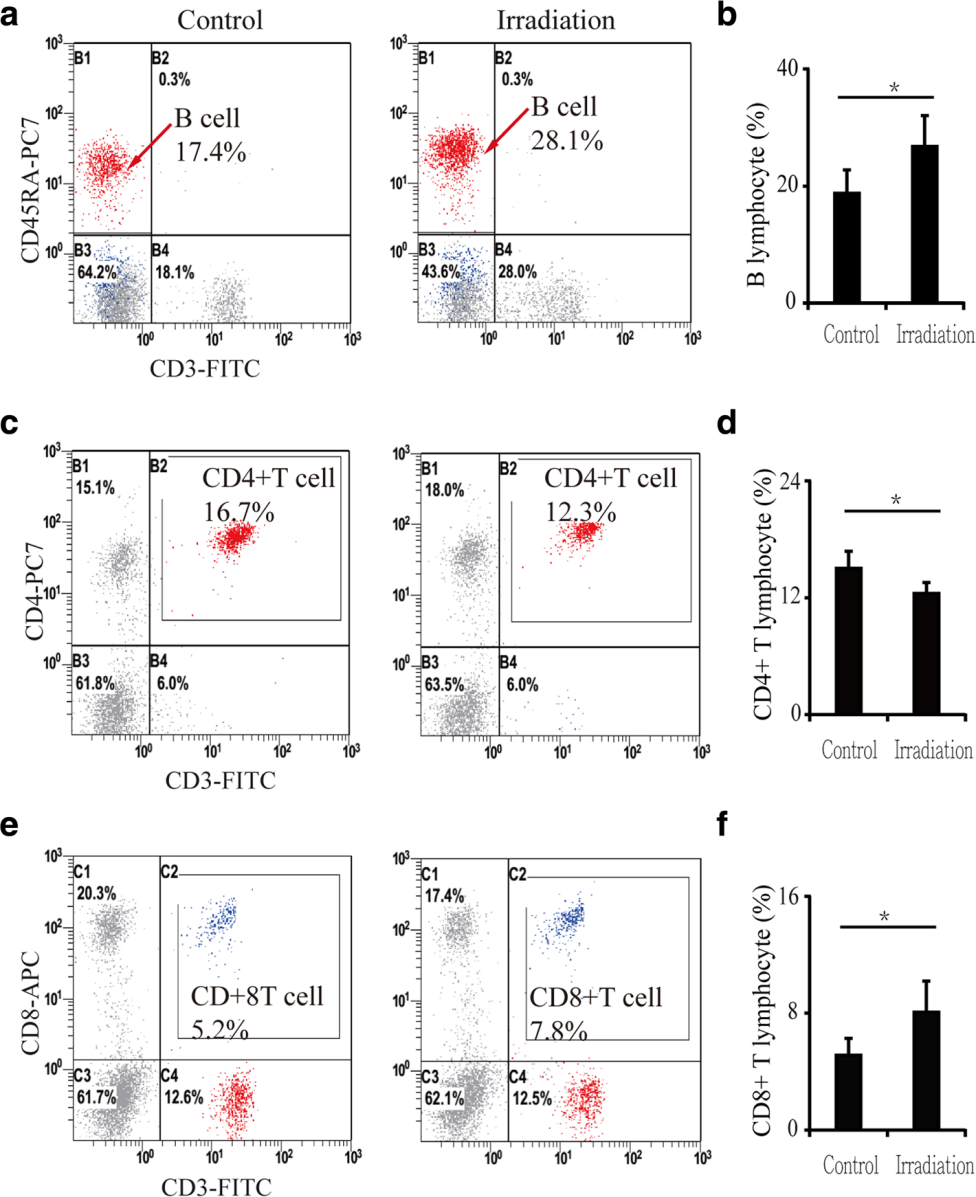
Lymphocytes in blood 12 weeks after irradiation detected by flow cytometry. **a** Flow cytometry of B lymphocytes; (**b**) B lymphocyte numbers increased in blood after irradiation. **c** Flow cytometry of CD4^+^ T lymphocytes; (**d**) CD4^+^ T lymphocyte numbers decreased in blood after irradiation. **e** Flow cytometry of CD8^+^ T lymphocytes; (**f**) CD8^+^ T lymphocyte numbers increased in blood after irradiation. Data are presented as the means ± standard deviations; ^*^ *P* < 0.05 (*n* = 6/group)

**Fig. 5:**
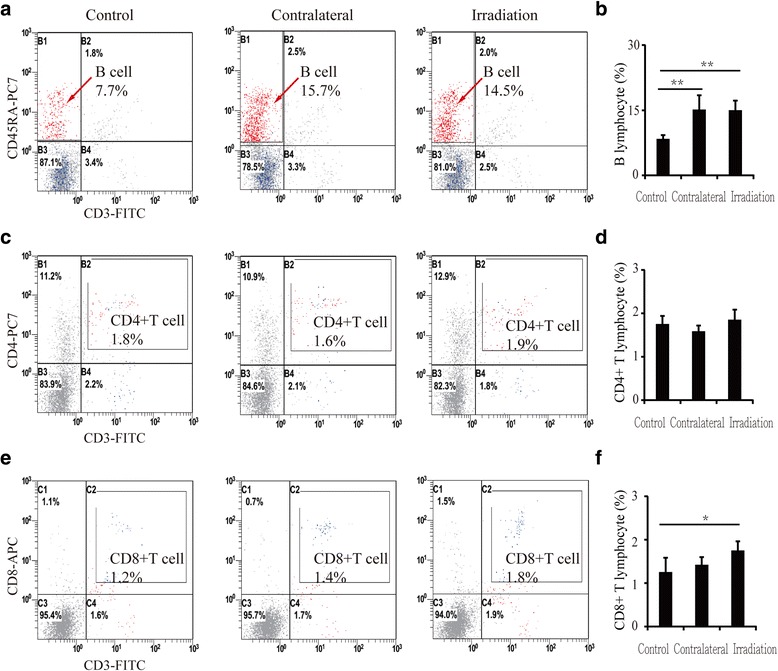
Lymphocytes in bone marrow 12 weeks after irradiation detected by flow cytometry. **a** Flow cytometry of B lymphocytes; (**b**) B lymphocyte numbers increased in contralateral and irradiated bone. **c** Flow cytometry of CD4^+^ T lymphocytes; (**d**) CD4^+^ T lymphocyte numbers exhibited no changes in bone marrow after irradiation. (**e**) Flow cytometry of CD8^+^ T lymphocytes; (**f**) CD8^+^ T lymphocyte numbers increased in contralateral and irradiated bone after irradiation. Data are presented as the means ± standard deviations; ^*^ *P* < 0.05 and ^**^ *P* < 0.01 (*n* = 6/group)

The numbers of lymphocytes in peripheral blood changed significantly after irradiation. B lymphocytes increased by 42.7% (*P* < 0.05) (Fig. 4a and b), CD4^+^ T lymphocytes decreased by 17.3% (*P* < 0.05) (Fig. 4c and d), and CD8^+^ T lymphocytes increased by 57.6% (*P* < 0.05) (Fig. 4e and f) relative to controls. Similarly, the number of lymphocytes in bone marrow was also altered: B lymphocytes increased by 82.6% (*P* < 0.01) in contralateral bone and by 80.7% (*P* < 0.01) in irradiated bone relative to controls (Fig. 5a and b). However, CD4^+^ T lymphocytes showed no significant changes between contralateral, irradiated and control bone (Fig. 5c and d). Meanwhile, CD8^+^ T lymphocytes increased slightly, but not significantly (13.5%, *P* > 0.05), in contralateral bone and increased by 40.3% (*P* < 0.05) in irradiated bone (Fig. 5e and f).

### Effect of lymphocytes from irradiated rats on the differentiation of BMSCs into osteoblasts/adipocytes in vitro

**Fig. 6:**
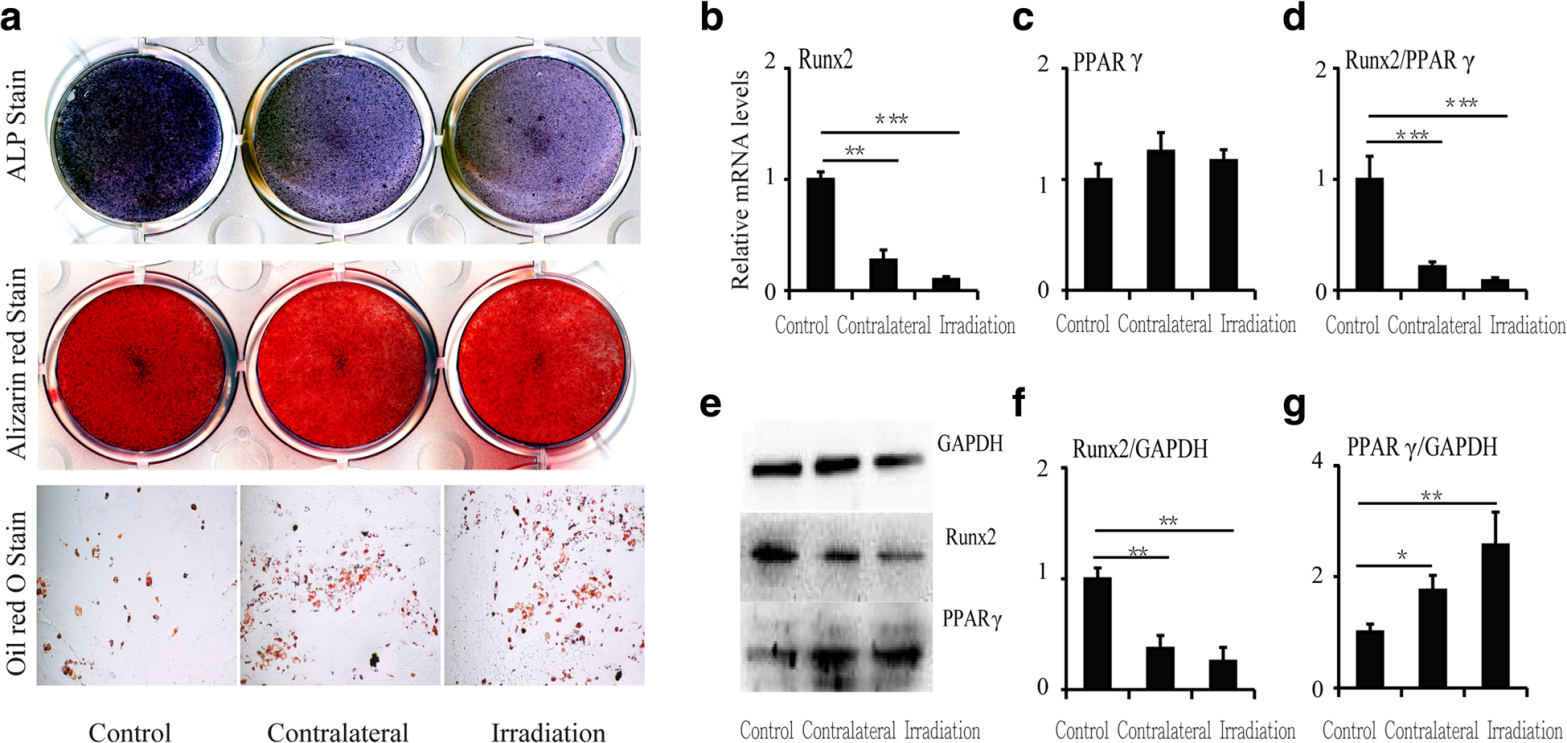
Changes in differentiation and relative mRNA and protein levels of BMSCs after co-culture with lymphocytes from irradiated rats. **a** ALP, Alizarin *Red*, and Oil *Red* O staining of co-cultured BMSCs. **b** RUNX2 mRNA expression, (**c**) PPARγ mRNA expression, and (**d**) RUNX2/PPARγ ratio. Western blot image (**e**) and RUNX2 (**f**) and PPARγ (**g**) protein expression levels at 12 weeks. Data are presented as the means ± standard deviations; ^*^ *P* < 0.05, ^**^ *P* < 0.01, and ^***^ *P* < 0.001 (*n* = 6/group). *ALP* alkaline phosphatase, *PPARγ* peroxisome proliferator-activated receptor γ, *RUNX2* runt-related transcription factor 2

After co-culture with lymphocytes from contralateral and irradiated bone marrow, the potential of BMSCs to differentiate into osteoblasts clearly decreased. ALP staining showed markedly decreased ALP-positive osteoblasts in co-cultures with lymphocytes from contralateral and irradiated sites relative to controls, and Alizarin Red staining showed the same trend. In contrast, Oil Red O staining showed increased adipocytes after co-culture with lymphocytes from irradiated rats, whether from irradiated or contralateral sites (Fig. 6a). RUNX2 RNA expression decreased significantly in BMSCs after co-culture, showing a 72.9% (*P* < 0.01) reduction in co-cultures with contralateral lymphocytes and a 97.2% (*P* < 0.001) reduction in co-cultures with irradiated lymphocytes (Fig. 6b). RUNX2 protein expression showed the same trend (Fig. 6e and f). PPARγ RNA expression increased only slightly (Fig. 6c); however, PPARγ protein expression was increased by 42.8% (*P* < 0.05) in co-cultures with lymphocytes from contralateral sites and by 60.9% (*P* < 0.01) in co-cultures with lymphocytes from irradiated sites (Fig. 6e and g).

## Discussion

Radiation-induced bone loss has always occurred in patients who undergo radiotherapy. Our study provides further evidence that losses in trabecular bone volume and microstructure occur at 12 weeks post-irradiation. Furthermore, bone loss was detected in our model at sites outside of the field of irradiation as well as at the site of irradiation. In view of the close relationship between the immune and skeletal systems [17], we investigated changes in B cells and CD4^+^ and CD8^+^ T lymphocytes in blood and bone marrow after focal irradiation. The results showed an increase in B lymphocytes and CD8^+^ T lymphocytes in both blood and bone marrow and a decrease in CD4^+^ T lymphocytes in blood. This phenomenon revealed that dysregulation of immune cells was not isolated to only irradiated areas, but rather was systemic.

Lymphocytes play a negative role in ovariectomy- and arthritis-induced osteoporosis by producing pro-inflammatory cytokines and receptor activator of nuclear factor kappa-B ligand (RANKL), thereby inducing osteoclasts and inhibiting osteoblasts [21–23]. Additionally, T cell expansion led to stimulation of osteoclastogenesis in ovariectomized mice [24–26]. The crucial role of immune organs in bone loss due to oestrogen deficiency has been well illustrated in thymectomized mice [25], in which bone loss induced by ovariectomy was attenuated by thymectomy. Recent data have also shown that B cells are the dominant producers of osteoprotegerin in the bone microenvironment in vivo [15]. B cell knockout mice have an osteoporotic phenotype with enhanced osteoclastic bone resorption, and reconstitution with B cells by adoptive transfer completely rescues mice from the development of osteoporosis and normalizes osteoprotegerin production [15]. Thus, B cells are very important for bone homeostasis. In contrast, it has been shown that activated B cells overexpress RANKL, contributing to bone resorption [16, 27], and that ovariectomy in mice increases the number of RANKL-expressing B lymphocytes in bone marrow [28]. A recent article showed that mice lacking RANKL in B cells were partially protected from ovariectomy-induced loss of cancerous bone [29]. The role of B lymphocytes has also been evaluated in diseases characterized by focal bone loss, such as periodontal inflammation [14, 30, 31] and rheumatoid arthritis [16, 32]. In rheumatoid arthritis, a recent study showed that B cell depletion ameliorates suppressed bone turnover [29]. This research suggests that normal lymphocytes are very important for maintaining bone homeostasis, but activated lymphocytes will lead to bone loss. However, many of these studies focus on the activity of lymphocytes on osteoclastogenesis. One possible reason we did not also find an increase in osteoclastogenesis in our experiment may be the detection time. A rapid increase in osteoclastogenesis has been previously reported 3 days after total-body radiation exposure [33, 34]. However, in our study, abnormal lymphocytes were still present throughout the body at 12 weeks after focal irradiation, and these abnormal lymphocytes were certainly involved in other pathological processes. A recent study showed that acute lymphoblastic leukaemia arising from the malignant proliferation of lymphoid precursors inhibited the differentiation of BMSCs into osteoblasts [35]. It has also been reported that dexamethasone (which suppresses lymphocytes) increased trabecular bone tissue in normal and ovariectomized mice, but nonsteroidal anti-inflammatory drugs (NSAIDs) (which do not suppress lymphocytes) did not [36]. This suggests that lymphocytes may be involved in bone formation.

In our study, we also found that low levels of serum OCN after focal irradiation indicated a decrease in bone formation; meanwhile, the number of adipocytes increased sharply in bone marrow. It is known that BMSCs can differentiate into either osteoblasts or adipocytes by respectively activating key genes such as RUNX2 or PPARγ [37, 38]. Therefore, we evaluated RUNX2 and PPARγ mRNA and protein levels in BMSCs after irradiation. The results revealed a sharp decrease in RUNX2 and a significant increase in PPARγ. Thus, radiation-induced damage changes the balance of differentiation in BMSCs, resulting in reduced osteogenic potential and increased adipogenic potential [39–46]. Most importantly, these changes in BMSCs were detected at sites outside of the field of irradiation as well as at sites of irradiation, demonstrating that systemic damage to BMSCs occurs after irradiation. In view of the persistent nature of abnormal lymphocytes in the body after focal irradiation and the negative effect on BMSCs of lymphocytes from acute lymphoid leukaemia, we co-cultured lymphocytes from irradiated rats with normal BMSCs. The co-cultured BMSCs had decreased RUNX2 expression and increased PPARγ expression, which upregulated the differentiation of BMSCs into adipocytes and downregulated their differentiation into osteoblasts. Thus, lymphocytes exerted a negative effect on bone formation by changing the differentiation potential of BMSCs over time after focal irradiation. In our previous study, we also tested inflammatory factors and oxidative stress reactions at 12 weeks after irradiation; no obvious changes were observed, and these results are in agreement with those of another recent report [47].

## Conclusions

Focal irradiation induced local and systemic bone trabecular loss. Subsequent increases in B cells and CD8^+^ lymphocytes regulated systemic bone loss by decreasing BMSC osteogenesis. This study also revealed the importance of lymphocytes in bone homeostasis over time after focal irradiation, which may offer new insight into the prevention and treatment of bone complications in patients who undergo radiotherapy, especially patients who require stem cell transplantation.

## Abbreviations

ALP: Alkaline phosphatase
BMSCs: Bone mesenchymal stem cells
BS/BV: Ratio of bone surface to the bone volume
BV/TV: Bone volume fraction, ratio of the segmented bone volume to the total volume of the region of the region of interest
CT: Computed tomography
Ct.Ar./Tt.Ar.: Cortical area fraction
Ct.Th: Average cortical thickness
OB.S/BS: Osteoblast surface to bone surface
OC.S/BS: TRAP-positive osteoclast surface to bone surface
OCN: Osteocalcin
PPARγ: Peroxisome proliferator-activated receptor γ
RANKL: Receptor activator of nuclear factor kappa-B ligand
RUNX2: Runt-related transcription factor 2
Tb.N: Trabecular number, measure of the average number of trabeculae per unit length
Tb/Th: Trabecular thickness, mean thickness of trabeculae
tBMD: Trabecular bone mineral density
TRAP5b: Tartrate-resistant acid phosphatase 5b
